# Association of Mild Thyroid Dysfunction and Adverse Prognosis Among Chinese Patients With Acute ST Segment Elevation Myocardial Infarction

**DOI:** 10.3389/fendo.2022.879443

**Published:** 2022-04-28

**Authors:** Mei-Fang Li, Ze-Tao Wei, Shuai Li, Qi-Ming Feng, Jing-Bo Li

**Affiliations:** ^1^Department of Emergency, Shanghai Jiao Tong University Affiliated Sixth People’s Hospital, Shanghai, China; ^2^Department of Emergency, Dan Zhou People’s Hospital, Dan Zhou, China; ^3^Department of Cardiology, Shanghai Jiao Tong University Affiliated Sixth People’s Hospital, Shanghai, China

**Keywords:** mild thyroid dysfunction, subclinical hypothyroidism, subclinical hyperthyroidism, low T3 syndrome, acute myocardial infarction, ST segment elevation myocardial infarction

## Abstract

**Aims:**

Thyroid hormones widely affect the cardiovascular system, but the effects of mild thyroid dysfunction on the clinical prognosis of patients with acute ST segment elevation myocardial infarction (STEMI) remains unclear. Our aims were to analyze the relations between mild thyroid dysfunction at admission and clinical outcomes in Chinese patients with STEMI.

**Methods:**

A total of 1,176 STEMI patients with the available data of thyroid function and follow-up were analyzed, including 348 patients with mild thyroid dysfunction [subclinical hypothyroidism (n=81), hyperthyroidism (SHyper) (n=51), and low triiodothyronine syndrome (LT3S) (n=216)] and 828 patients with euthyroid function. During a median 4.4-year follow-up, in-hospital mortality, cardiac and all-cause mortalities were subsequently compared among the four groups.

**Results:**

Compared with the euthyroid group, STEMI patients in the SHyper and LT3S groups faced obviously increased risks of in-hospital death [odds ratio (OR): 5.007, 95% confidence interval (CI): 1.246–20.124, p = 0.023 and OR: 2.491, 95% CI: 1.054–5.887, p = 0.037, respectively) even after adjustment for various confounding factors. During a median 4.4-year follow-up, STEMI patients with LT3S at baseline had higher cardiovascular mortality [hazard ratio (HR): 1.880, 95% CI: 1.178–2.998, p = 0.008] and all-cause mortality HR: 1.647, 95% CI: 1.072–2.531, p = 0.023] than those with euthyroid at baseline, whereas no significantly increased mortality was found for STEMI patients with SCH and SHyper at baseline.

**Conclusions:**

STEMI patients with SHyper at admission had increased risk of in-hospital mortality, and STEMI patients with LT3S at baseline had worse prognosis and higher incidences of in-hospital mortality and cardiovascular and all-cause deaths compared with euthyroid patients.

## Introduction

Acute myocardial infarction (AMI) is caused by a variety of factors and remains at a high rate of mortality, even though great progresses are made in pharmacotherapy and myocardial reperfusion (1, 2). In the neuroendocrine systems, thyroid hormones play fundamental roles in cardiovascular homeostasis by regulating the heart rate, cardiac contractility, and arterial peripheral resistance. Several observational studies have also shown that mild thyroid dysfunction, including subclinical hypothyroidism (SCH), subclinical hyperthyroidism (SHyper), and low T3 syndrome (LT3S), is quite common in AMI patients (3, 4). Recently, the harmful effects of overt thyroid dysfunctions on the cardiovascular system have been well established in both the general populations and cardiac patients (5, 6). However, conclusions on the associations of mild thyroid dysfunction and adverse outcomes are still controversial and related studies mainly focus on heterogeneous patients with various cardiac diseases. For example, some studies discovered that SCH/SHyper were related with higher risks of coronary heart disease (CHD) and mortality (7, 8), while others found that SCH/SHyper did not cause adverse cardiovascular outcomes (9, 10). In addition, the impacts of mild thyroid dysfunction on the mortality of AMI patients also remain unclear.

Furthermore, the studies regarding the influences of mild thyroid dysfunction on the poor prognosis in Chinese patients suffering from AMI are also extremely limited. One of our aims was to compare and assess the effects of mild thyroid dysfunction on cardiac function and in-hospital mortality in Chinese patients with acute ST segment elevation myocardial infarction (STEMI). Moreover, we also explored and evaluated the impacts of mild thyroid dysfunctional states at baseline on the cardiovascular and all-cause mortality rates during a median follow-up period of 4.4 years.

## Materials and Methods

### Study Population

A total of 1,847 Chinese AMI patients who were admitted to the Department of Cardiology in Shanghai Jiao Tong University Affiliated Sixth People’s Hospital during the period from September 2007 to September 2014 were enrolled in the present study. Among them, 671 candidates were successively eliminated due to the following reasons: (1) patients with non-ST-elevation AMI (NSTEMI) (n=95); (2) known or clinically thyroid disorders (n=76); (3) current or previous treatment with thyroid hormone supplementation, antithyroid medications, corticosteroids, dopamine, dobutamine, amiodarone or lithium (n=52); (4) thyroid indicators were obtained after coronary angiography or CTA (n=239); (5) unable to complete coronary examination due to end-stage diseases (n=83);and (6) a lack of clinical data or loss of follow-up (n=126). Ultimately, 1,176 participants took part in this analysis and then they were divided into four groups including euthyroidism, SCH, SHyper, and LT3S according to their thyroid hormone values. During a median 4.4-year follow-up, we subsequently made comparisons on in-hospital mortality, cardiac and all-cause mortalities among the four groups. Our study was approved by the ethics committee of the Shanghai Jiao Tong University Affiliated Sixth People’s Hospital, and all participants signed written informed consent forms.

### Physical Examination and Laboratory Measurements

The physical and laboratory examinations in this study were collected by well-trained physicians. Briefly, height, weight, blood pressure, and heart rate (HR) were recorded and detailed information on the history of diabetes, hypertension, alcohol use, and smoking habits was collected through a standard interview when the patients entered into the Department of Cardiology. Body mass index (BMI) was obtained as weight divided by the square of height. Thyroid profile including free triiodothyronine (FT3), free thyroxine (FT4), thyroid-stimulating hormone (TSH), and other blood indicators such as white blood cell (WBC), hemoglobin, C-reactive protein (CRP), serum creatinine (SCr), serum albumin, total cholesterol (TC), total triglyceride (TTG), high-density lipoprotein cholesterol (HDL-C), low-density lipoprotein cholesterol (LDL-C), fasting plasma glucose (FPG), N-terminal pro-B-type natriuretic peptide (NT-proBNP), and troponin I (TnI) were obtained from blood samplings after an overnight fast within 24 h after admission and prior to coronary angiography or CTA. The thyroid function profile was gathered using a chemiluminescence technique (Cobas 6000; Roche Diagnostics GmbH, Mannheim, Germany). The estimated glomerular filtration rate (eGFR) was obtained by the simplified MDRD formula: eGFR = 186.3 × (serum creatinine)^−1.154^ × (age)^−0.203^ (× 0.742 if woman) (11).

### Coronary Artery Examination and Follow-Up

All patients underwent coronary angiography or CTA to make a definitive diagnosis, and the culprit vessels were treated by oral medications, percutaneous coronary intervention, or coronary artery bypass graft (1, 12). The echocardiography was performed by experienced ultrasonographers, and left ventricular ejection fraction (LVEF) was recorded *via* an Acuson Sequoia 512 scanner with a probe of 5-13-MHz following a standard protocol. The data regarding Killip class, revascularization (percutaneous coronary intervention or coronary artery bypass graft), medications at discharge, and in-hospital deaths were obtained from their discharge summaries. After the discharge from hospital, a regular clinical follow-up was conducted through telephone or office visits annually.

### Diagnostic Criteria and Outcomes

AMI was diagnosed when chest pain for more than 30 min with dynamic 12-lead electrocardiogram (ECG) changes or elevated troponin enzymes and STEMI were defined as AMI accompanied by ST segment elevation in ≥2 contiguous ECG leads according to the ACC/AHA guidelines (1). Severe acute heart failure was regarded as Killip class > II (13). The reference ranges of thyroid function in our hospital were as follows: FT3 3.1–6.8 pmol/L, FT4 12.0–22.0 pmol/L, and TSH 0.27–4.20 mIU/l, respectively. Euthyroidism was identified as the levels of TSH, FT4, and FT3 within their respective reference ranges. SCH was determined by a TSH level above 4.20 mIU/l with a normal FT4 level. SHyper was regarded as TSH < 0.27 mIU/l with normal FT3 and FT4 levels. LT3S was defined when FT3 < 3.1 pmol/L with normal TSH and FT4 levels.

Accidental death was excluded, and all deaths were caused by any natural factor. Cardiovascular death was defined as the mortality attributable to myocardial infarction, cardiogenic shock, significant arrhythmia, progressive heart failure, or pulmonary embolism without a precipitating factor. Sudden unexpected death outside the hospital was regarded as a cardiac death, and no autopsy was performed. In-hospital deaths were not included into all-cause mortality and cardiovascular death. All events were identified and sorted by two cardiologists. We calculated the survival times from the date of the STEMI to the date of death.

### Statistical Analyses

Data were analyzed by SPSS 19.0 software. Firstly, normality was checked for continuous variables by Q-Q plots. Normally distributed variables were expressed as mean ± standard deviation and were compared using one-way ANOVA with LSD, whereas unevenly distributed variables were represented as median with interquartile range (IQR) and were compared by the Kruskal–Wallis test. Secondly, categorical variables were expressed as absolute numbers (percentages) and were compared by the χ2 test. Thirdly, three binary logistic regression models were used to assess the association of mild thyroid dysfunction and in-hospital mortality: a non-adjusted model; an age- and sex-adjusted model; and a multivariable model that included all variables with p-value < 0.05 from the univariate analyses through the forward stepwise procedure. The results were expressed as odds ratios (ORs) with associated 95% confidence intervals (CIs). Fourthly, the univariate, age-, and sex-adjusted and multivariate Cox regression analyses were performed to analyze the effects of mild thyroid dysfunction states on cardiovascular and all-cause mortality. All baseline variables with p-value <0.05 in univariate analyses were entered into the multivariate Cox regression analysis and analyzed by forward stepwise regression. Results were reported as hazard ratios (HRs) with associated 95% CIs. The cumulative survival rates were described by Kaplan–Meier curves and were compared between groups by the log rank test based on the euthyroid group as the reference group. P < 0.05 was considered as statistically significant.

## Results

### Baseline Characteristics of Studied Subjects

Of the 1,176 participants analyzed, 828 patients (70.4%) were euthyroid, 81 patients (6.9%) had SCH, 51 patients (4.3%) had SHyper, and 216 patients (18.4%) had LT3S. The baseline demographic and clinical characteristics of these four groups are displayed in **Table 1**. Individuals in the SCH and LT3S groups tended to be older and women and had lower LDL-C, hemoglobin and eGFR and less smoking and revascularization, as well as higher SCr and CRP compared with the euthyroid and SHyper groups. In addition, the prevalence of diabetes mellitus and hypertension, BMI, SBP, DBP, HR, FT3, TSH, FPG, serum albumin, WBC, and discharge medical therapy (angiotensin-converting enzyme inhibitor/angiotensin II receptor blocker, diuretics) were also significantly different among the four groups (all p < 0.05).

**Table 1:** Baseline characteristics of study population by mild thyroid dysfunctional states.

Variables	Euthyroid (n=828)	SCH group (n=81)	SHyper group (n=51)	LT3S group(n=216)	p-value
^a^Age (years)	70 (57-78)	76 (69-82)	63 (54-73)	76 (66-82)	<0.001
Male (n,%)	573 (69.2%)	42 (51.9%)	44 (86.3%)	130 (60.2%)	<0.001
Diabetes mellitus (n,%)	244 (29.5%)	27 (33.3%)	4 (7.8%)	83 (38.4%)	<0.001
Hypertension (n,%)	533 (64.4%)	53 (65.4%)	18 (35.3%)	141 (65.3%)	<0.001
Prior PCI or CABG (n,%)	24 (2.9%)	1 (1.2%)	2 (3.9%)	5 (2.3%)	0.756
Smoking (n,%)	505 (61%)	35 (43.2%)	39 (76.5%)	120 (55.6%)	0.001
Alcohol (n,%)	48 (5.8%)	3 (3.7%)	3 (5.9%)	8 (3.7%)	0.571
BMI (kg/m^2^)	24.10 ± 2.19	23.52 ± 2.60	23.93 ± 1.49	23.41 ± 2.54	<0.001
Vital signs and laboratory tests at admission					
SBP (mmHg)	130 ± 22	130 ± 23	119 ± 19	125 ± 25	0.002
DBP (mmHg)	75 ± 13	73 ± 13	72 ± 13	71 ± 14	0.005
^a^HR (beats/min)	75 (60-82)	82 (65-89)	83 (62-92)	80 (61-89)	<0.001
^a^FT3 (pmol/L)	3.92 (3.60-4.37)	3.80 (3.35-4.18)	3.80 (3.50-4.10)	2.70 (2.36-2.90)	<0.001
FT4 (pmol/L)	16.07 ± 2.27	16.05 ± 2.40	15.78 ± 2.30	15.81 ± 2.36	0.429
^a^TSH (mIU/L)	1.32 (0.78-2.04)	5.53 (4.66-6.89)	0.21 (0.15-0.23)	1.11 (0.61-1.82)	<0.001
^a^FPG (mmol/L)	5.99 (5.28-7.48)	6.35 (5.39-7.84)	6.54 (5.71-7.78)	6.69 (5.60-8.76)	0.001
TC (mmol/L)	4.55 ± 1.15	4.26 ± 1.10	4.64 ± 1.27	4.43 ± 1.19	0.111
TTG (mmol/L)	1.51 ± 0.89	1.55 ± 0.83	1.42 ± 0.53	1.34 ± 0.99	0.095
HDL-C (mmol/L)	1.13 ± 0.24	1.05 ± 0.30	1.10 ± 0.32	1.08 ± 0.28	0.861
LDL-C (mmol/L)	2.96 ± 1.03	2.67 ± 0.96	3.13 ± 1.11	2.78 ± 1.00	0.011
^a^SCr (μmol/L)	83 (69-101)	99 (75-130)	83 (72-93)	98 (75-136)	<0.001
eGFR (ml/min/1.73 m^2^)	76.78 ± 33.79	60.93 ± 29.12	82.97 ± 32.72	61.31 ± 30.97	<0.001
Serum albumin (g/L)	41 ± 6	40 ± 5	40 ± 4	38 ± 6	<0.001
^a^WBC (×10^9^/L)	7.8 (6.1-10.2)	7.2 (5.6-9.1)	8.4 (6.5-11.9)	9.4 (6.8-12.5)	<0.001
^a^Hemoglobin (g/L)	139 (126-143)	123 (115-138)	141 (136-143)	126 (114-133)	<0.001
^a^CRP (mg/L)	3.4 (1.8-7.2)	4.9 (2.6-12.4)	3.4 (2.8-20.7)	11.6 (5.8-29.7)	<0.001
Revascularization (n,%)	587 (70.9%)	43 (53.1%)	42 (82.4%)	119 (55.1%)	<0.001
Discharge medical therapy					
Aspirin (n,%)	801 (99.9%)	75 (100%)	46 (100%)	185 (100%)	0.858
Clopidogrel/Ticagrelor (n,%)	796 (99.1%)	75 (100%)	46 (100%)	185 (100%)	0.443
Statin (n,%)	799 (99.5%)	75 (100%)	46 (100%)	185 (100%)	0.675
β-receptor blocker (n,%)	471 (58.7%)	54 (72%)	27 (58.7%)	110 (59.5%)	0.173
ACEI/ARB (n,%)	413 (51.4%)	31 (41.3%)	30 (65.2%)	104 (56.2%)	0.034
Diuretic (n,%)	564 (70.2%)	45 (60%)	38 (82.6%)	147 (79.5%)	0.002

PCI, percutaneous coronary intervention; CABG, coronary artery bypass graft; BMI, body mass index; SBP, systolic blood pressure; DBP, diastolic blood pressure; HR, heart rate; FT3, free triiodothyronine; FT4, free thyroxine; TSH, thyroid-stimulating hormone; FPG, fasting plasma glucose; TC, total cholesterol; LDL-C, low-density lipoprotein cholesterol; HDL-C, high-density lipoprotein cholesterol; TTG, total Triglyceride; SCr, serum creatinine; eGFR, estimated glomerular filtration rate; WBC, white blood cell; CRP, C-reactive protein; ACEI, angiotensin-converting enzyme inhibitor; ARB, angiotensin II receptor blocker.

Continuous variables were expressed as mean ± standard deviation or median with interquartile range, while categorical variables were expressed as percentages.

a Non-normal distribution of continuous variables.

### Comparison of Myocardial Injury and Cardiac Dysfunction Among the Four Groups at Baseline

**Figure 1:**
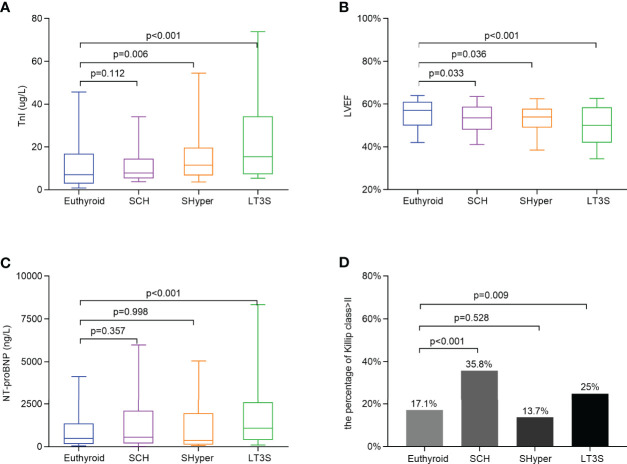
Comparison of myocardial injury and cardiac dysfunction by mild thyroid dysfunction status at baseline. **(A)** Comparison of the TnI levels among the four groups. **(B)** Comparison of the LVEF among the four groups. **(C)** Comparison of NT-proBNP levels among the four groups. **(D)** Comparison of the percentage of Killip class > II among the four groups. Data are shown as the median with 10th and 90th percentiles.

A comparison of myocardial injury and cardiac dysfunction among the four groups is shown in **Figure 1**. Compared with the subjects in the euthyroid group, the value of TnI for STEMI patients in the SHyper and LT3S groups was significantly higher (11.50 [IQR 6.73-19.68] ug/L and 15.43 [IQR 7.28-34.30] ug/L versus 7.04 [IQR 2.93-16.89] ug/L, respectively) (**Figure 1A**). The LVEF for STEMI patients in the SCH, SHyper, and LT3S groups was remarkably lower than that in the euthyroid group (54 [IQR 48-59] %, 54 [IQR 49-58] %, and 50 [IQR 42-59] % vs. 57 [IQR 50-61] %, respectively) (**Figure 1B**). The percentage of Killip class > II for STEMI patients in the SCH and LT3S groups was also obviously higher than that in the euthyroid group (35.8% and 25% vs. 17.1%) (**Figure 1D**). In addition, only participants in the LT3S group had obviously higher levels of NT-proBNP (1091 [IQR 400-2,600]ng/L vs. 483.25 [IQR 169.9-1,371]ng/L, respectively) compared with those in euthyroid group (**Figure 1C**).

### Comparison of Mortality Rate Among the Four Groups

**Figure 2:**
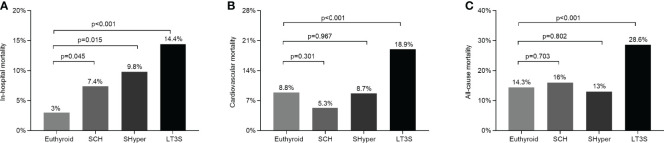
Comparison of in-hospital, cardiovascular, and all-cause mortality by mild thyroid dysfunction status. **(A)** Comparison of in-hospital mortality among the four groups. **(B)** Comparison of cardiovascular mortality among the four groups. **(C)** Comparisons of all-cause mortality among the four groups. Cardiovascular and overall mortality did not include in-hospital mortality.

The comparison of mortality rate among the four groups is displayed in **Figure 2**. Compared with the subjects in the euthyroid group, the STEMI patients in SCH, SHyper, and LT3S groups successively had significantly higher in-hospital mortality rate (7.4%, 9.8%, and 14.4% vs. 3%, respectively) (**Figure 2A**). During a median follow-up period of 4.4 (IQR2-6.1) years, 186 deaths occurred and 114 of them were caused by cardiovascular events. Compared to the euthyroid group, STEMI patients in the LT3S group at baseline had remarkably higher cardiovascular and all-cause mortality rates (18.9% vs. 8.8%, and 28.6% vs. 14.3%, respectively), whereas those STEMI patients in the SCH and SHyper groups at baseline did not exhibit significant discrepancies on the long-term mortality rate (**Figures 2B, C**).

### Association of Mild Thyroid Dysfunction and Short- and Long-Term Mortality Risks

**Table 2** presents the comparison of in-hospital mortality among mild thyroid dysfunction *via* binary logistic regression analyses. The SCH group exhibited a remarkably higher risk of in-hospital death than the euthyroid group in the non-adjusted model, but the significant association disappeared after adding other confounders. Additionally, given the euthyroid group as a reference, the SHyper group and LT3S group showed an obviously increased risk for in-hospital death (OR: 5.007, 95%CI: 1.246–20.124, p = 0.023 and OR: 2.491, 95%CI: 1.054 to 5.887, p = 0.037, respectively) even after adjusting for various confounding factors.

**Table 2:** Comparison of in-hospital mortality among mild thyroid dysfunction status.

	Mortality	Univariate model			Age- and sex-adjusted model			Multivariate model^*^		
	n (%)	OR	95%CI	p-value	OR	95%CI	p-value	OR	95%CI	p-value
**Euthyroid**	25 (3%)	**1 [reference]**			**1 [reference]**			**1 [reference]**		
**SCH**	6 (7.4%)	2.570	1.022-6.460	0.045	1.978	0.772-5.072	0.155	2.086	0.540-8.057	0.286
**SHyper**	5 (9.8%)	3.491	1.278-9.539	0.015	3.928	1.407-10.969	0.009	5.007	1.246-20.124	0.023
**LT3S**	31(14.4%)	5.382	3.104-9.334	<0.001	4.083	2.309-7.221	<0.001	2.491	1.054-5.887	0.037

^*^Variables with p < 0.05 in univariate analysis [age, SBP, WBC, hemoglobin, serum albumin, FPG, eGFR, LVEF, NT-proBNP, and revascularization (PCI, CABG)] were included in the multivariate model.

NT-proBNP, N-terminal pro-B-type natriuretic peptide; LVEF, left ventricular ejection fraction.

**Figure 3:**
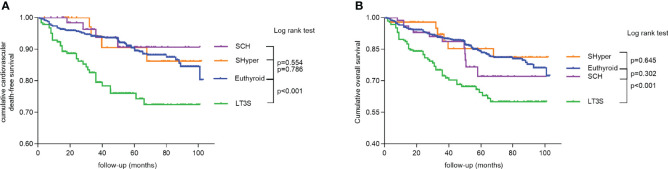
Kaplan–Meier curves for long-term survival to demonstrate the association of mild thyroid dysfunction status with mortality. **(A)** Cumulative cardiovascular death-free survival among the four groups. **(B)** Cumulative overall survival among the four groups.

**Table 3** shows the comparison of long-term cardiovascular and all-cause mortality among mild thyroid dysfunction at baseline by Cox proportional hazards analyses. After adjusting for covariates, a significantly increased risk of cardiac and all-cause mortalities was found in the LT3S group at baseline but not in the SCH or SHyper group at baseline. Accordingly, the risk of cardiovascular mortality in the LT3S state at baseline remained 1.880 folds (95%CI: 1.178–2.998; p = 0.008) and the risk of all-cause mortality in the LT3S state at baseline was still 1.647 folds (95%CI: 1.072–2.531; p = 0.023) using the euthyroid state at baseline as the reference, whereas STEMI patients in SCH and SHyper groups at baseline were not associated with increased cardiac and all-cause mortalities. The Kaplan–Meier analysis also demonstrated that the cardiovascular death-free survival and overall survival of STEMI patients in the LT3S group at baseline were obviously shorter than those in the euthyroid group at baseline (**Figure 3**).

**Table 3:** Comparison of cardiovascular and all-cause mortality among mild thyroid dysfunction status.

	Mortality	Univariate model			Age- and sex-adjusted model			Multivariate model		
	n (%)	OR	95%CI	p-value	OR	95%CI	p-value	OR	95%CI	p-value
**Cardiovascular mortality**										
**Euthyroid**	71 (8.8%)	**1 [reference]**			**1 [reference]**			**1 [reference]**^**a**^		
**SCH**	4 (5.3%)	0.723	0.264-1.981	0.528	0.564	0.205-1.551	0.267	0.277	0.067-1.150	0.077
**SHyper**	4 (8.7%)	0.897	0.327-2.459	0.833	1.286	0.467-3.539	0.626	0.871	0.270-2.808	0.817
**LT3S**	35 (18.9%)	2.688	1.791-4.035	<0.001	2.118	1.395-3.217	<0.001	1.880	1.178-2.998	0.008
**All-cause mortality**										
**Euthyroid**	115 (14.3%)	**1 [reference]**			**1 [reference]**			**1 [reference]**^**b**^		
**SCH**	12 (16%)	1.344	0.741-2.438	0.330	1.049	0.576-1.909	0.876	0.485	0.176-1.336	0.162
**SHyper**	6 (13%)	0.842	0.370-1.914	0.681	1.146	0.503-2.613	0.745	0.973	0.388-2.438	0.954
**LT3S**	53 (28.6%)	2.519	1.817-3.491	<0.001	2.006	1.437-2.800	<0.001	1.647	1.072-2.531	0.023

a Variables with p < 0.05 in univariate analysis [age, smoking, WBC, Hb, TC, TG, LDL-c, eGFR, CRP, LVEF, Killip class, and revascularization (PCI, CABG)] were entered into the multivariate model for cardiovascular mortality.

b Variables with p < 0.05 in univariate analysis [age, sex, smoking, diabetes, TnTI, WBC, Hb, Alb, TC, TG, LDL-c, eGFR, CRP, NT-proBNP, LVEF, Killip class, and revascularization (PCI, CABG)] were included in the multivariate model for all-cause mortality.

TnI, troponin I; NT-proBNP, N-terminal pro-B-type natriuretic peptide; LVEF, left ventricular ejection fraction.

## Discussion

In this prospective, single-center observational study, the impacts of mild thyroid dysfunction on in-hospital mortality, long-term cardiovascular and all-cause mortality were assessed in Chinese STEMI patients. Our results demonstrated that the STEMI patients with SHyper and LT3S faced a remarkably increased risk of in-hospital mortality in relation to euthyroid patients. During a long (median 4.4-year) follow-up, we found that LT3S at baseline was still associated with worse cardiovascular and all-cause mortality while SCH or SHyper at baseline did not affect the long-term prognosis of STEMI patients.

For AMI patients with SCH, the research on the associations of SCH and worse clinical outcomes was quite limited, although several observational studies suggested that elevated TSH beyond the normal range was a reliable marker for adverse outcome in AMI patients. For example, Zhu et al. (14) reported that increased TSH above the reference range was related to worse long-term prognosis and TSH > 3.5 mIU/L worked as an independent predictor for worse 2.5-year mortality in STEMI patients. Soeiro et al. (15) also found that acute coronary syndrome (ACS) patients with TSH > 4 mIU/L at admission had lower LVEF and faced more major adverse cardiac events but not mortality than those patients with TSH ≤ 4 mIU/L during hospitalization. However, both two studies above did not consider T3 and T4 when grouping and the elevated TSH group would have been divided into SCH and overt hypothyroidism subgroups if considered. Whether SCH and overt hypothyroidism alone are a risk factor for worse clinical outcomes in AMI patients arouses more interest, and it is also worth exploring. Recently, Seo et al. (16) showed that among AMI patients, the all-cause mortality was significantly higher in the elevated TSH group than that in the normal TSH group; whereas in the subgroup analysis, the SCH group was only remarkably correlated with all-cause mortality in model 1, but no significant differences were found in other 5 models after adding other confounding factors compared with the euthyroid group. The results of another prospective cohort study also displayed that there was no association between SCH and all-cause and cardiac mortality in Chinese patients with ACS undergoing percutaneous coronary intervention (PCI) after adjustment for confounders; however, ACS included AMI and unstable angina (17). Aligned with them, our present study found that SCH patients with STEMI exhibited significantly decreased LVEF rather than any mortality in comparison with the euthyroid patients in other models after adding other confounders, although SCH had an obviously higher risk of in-hospital death in univariate analysis. The present findings regarding LVEF were also confirmed by Pingitore and colleagues on the animal models of AMI (18), which showed the administration of thyroid hormone-enhanced myocardial remodeling and improved left ventricular function.

Different from us, a retrospective observational study by Izkhakov et al. (19) in STEMI patients undergoing PCI showed that SCH patients suffered from a higher incidence of poor in-hospital outcomes and short- and long-term mortality than euthyroid patients. The different basic characteristics of patients may help to explain the discrepancy between us, that is, the SCH patients in the study of Izkhakov et al. (19) were more likely to be men and smokers, while the SCH patients in our study were inclined to be women and non-smokers. Up to now, only a few studies assessed the specific association between SCH and mortality in patients with ischemic heart diseases and concluded contradictory results. The study of Izkhakov et al. (19) and two recent studies by Zhang et al. (20) and Lee et al. (21) reported that patients who were treated with PCI faced a higher risk of cardiovascular or all-cause mortality, while our present study and a large cohort study of older patients who were treated with PCI (22) showed that no associations were found between SCH and all-cause and cardiac deaths. The systematic review and meta-analysis of prospective cohort studies may help to explain the above difference, which showed that SCH had a stronger association with cardiovascular and all-cause mortality in individuals < 65 years than people ≥ 65 years (23). Additionally, the mean age in the studies of Izkhakov et al. (19), Zhang et al. (20), and Lee et al. (21) was successively 62, 64.6, and 66.2 years while the mean age in our present study and the cohort study of older patients (22) was 76 and 70.4 years, respectively. However, given the current few studies, the results on the prognostic significance of SCH in AMI patients need to be detailed interpretations and further verifications in the future studies.

With respect to the relations of SHyper with cardiovascular and total mortality, many studies have been made but with conflicting conclusions. For example, seven meta-analyses were found to discuss this issue so far. The third of them demonstrated that patients with SHyper faced a rising risk of total mortality and CHD mortality, particularly for those with suppressed TSH levels < 0.10 mIU/L (7, 8, 24), while other four studies did not (9, 10, 25, 26). Nevertheless, there is a lack of specific data on AMI as most of the above studies were mainly made in general population. Molinaro et al. (27) firstly found that SHyper was associated with an increased risk of cardiac and overall mortality in 1,026 patients with acute cardiac diseases during a 30-month follow-up, whereas only 285 of them were caused by AMI. In contrast, in a recent retrospective study with a median 2.5-year follow-up, no relation was observed between the decreased TSH and poor clinical outcomes in the population of STEMI; however, studied subjects were not further stratified by FT3 or FT4 in this study (14). The ThyrAMI-1 study also showed that AMI patients in the SHyper group did not alter all-cause mortality relative to those in the euthyroid group (28). Consistent with the above studies on AMI, our current study demonstrated that the SHyper group did not correlate with the risks of cardiovascular deaths and all-cause deaths compared with the euthyroid group during a median 4.4-year follow-up in patients with STEMI; however, we also found that the SHyper group had a significantly higher in-hospital mortality than the euthyroid group. Our findings suggested that SHyper patients at the early stage of AMI may tend to face the risk of short-term worse clinical implications, which should be given additional management strategy. Further, a large scale of prospective cohorts is needed to verify these findings.

Recently, a systematic review and meta-analysis discovered that the prevalence of LT3S in heart failure (24.5%), myocardial infarction (18.9%), and acute coronary syndrome (17.1%) is quite high (29). Similar to this, the rate of LT3S in our present study was 18.4% among Chinese STEMI patients. In our study, we found that STEMI patients with LT3S had more serious myocardial injury that was diagnosed by higher TnI and more severe cardiac dysfunction that was assessed by lower LVEF and higher NT-proBNP compared with the euthyroid patients, which was in line with previous studies (30, 31) and suggested that LT3S was correlated with the severity of AMI. In addition, accumulating evidence has supported the hypothesis of the role for LT3S in the prognosis of AMI patients. For example, clinical studies in some developed countries have reported that the prognosis of AMI patients with LT3S was significantly worse than those AMI patients with euthyroid functions, independent of other risk factors (32, 33). Nevertheless, thyroid dysfunction and the occurrences of heart diseases changed with ethnicities (34, 35), and the rates of reperfusion therapy in China were lower than those in western countries (36, 37). Therefore, it is extremely important to clarify the relations between LT3S and the prognosis of Chinese AMI patients as limited studies were made in Chinese AMI patients by far. Su et al. (38) explored that patients with LT3S faced a remarkably higher in-hospital cardiovascular death rate than those without LT3S; however, long-term outcomes were not conducted in their studies. Zhang et al. (31) and Song et al. (39) discovered that independent associations existed between low fT3 levels and 30-day and 1-year all-cause deaths in Chinese AMI patients; however, the TSH and FT4 were not considered when grouping and the periods of follow-up seemed to be relatively short. In line with them, our present study further displayed that STEMI patients in the LT3S group had a remarkably higher in-hospital mortality rate and obviously higher incidences of cardiovascular and all-cause deaths during a relatively long (4.4-year) follow-up compared with the euthyroid group, which was verified by the multivariate Cox proportional hazard regression analyses. The above conclusions and our results indicated that LT3S may be a reliable marker of adverse clinical results for AMI patients and may increase the predictive power of current risk core models in the future clinical practice.

Several limitations should be mentioned. Firstly, the objects who took part in our study were STEMI and from a single center of Chinese Han population, which may confine the generalizability of our results to patients with NSTEMI and other ethnic groups. Secondly, a thyroid function test was performed only before coronary angiography or CTA without tracking follow-up, but all samples were collected in the morning to avoid a circadian variation of thyroid hormones (40). Thirdly, given the primary purpose of our study and limited number of patients with mild thyroid dysfunction in our study, we think that our results would have been more valuable if the studied objects in our study were further stratified by age or revascularization. Therefore, more multicenter studies may need to validate our findings and further evaluate whether an altered thyroid function or treatment of thyroid dysfunction could help to improve the clinical outcomes.

## Conclusions

Our results suggested that SHyper may be a risk factor for in-hospital deaths in STEMI patients. Furthermore, LT3S may be considered as a prognostic indicator for poor short- and long-term mortality. The current findings indicate that a routine testing of thyroid function prior to coronary angiography or CTA should be recommended and is highly valued to help identify and administer AMI patients at high risk of adverse events and deaths. Further studies are needed to evaluate the additional role of mild thyroid dysfunction in a prognostic algorithm of AMI severity and whether thyroid replacement therapy lowers mortality in AMI patients.

## Data Availability Statement

The original contributions presented in the study are included in the article/supplementary material. Further inquiries can be directed to the corresponding authors.

## Ethics Statement

The studies involving human participants were reviewed and approved by Shanghai Jiao Tong University Affiliated Sixth People’s Hospital. The patients/participants provided their written informed consent to participate in this study.

## Author Contributions

Q-MF and J-BL designed the study, revised and reviewed the manuscript. M-FL, Z-TW and SL collected clinical data and follow-up. M-FL and Z-TW worked together, performed statistical analysis and wrote the manuscript. All authors edited the manuscript and approved the final manuscript.

## Funding

The authors declare that this study received fundings from the National Natural Science Foundation of China (grant numbers 81502316), the Translational Medicine National Key Science and Technology Infrastructure Open Project (grant number TMSK-2021-116), and the Exploratory Clinical Research Project of Shanghai Jiao Tong University Affiliated Sixth People’s Hospital (grant number ynts202105). The funders were not involved in the study design, collection, analysis, interpretation of data, the writing of this article or the decision to submit it for publication.

## Conflict of Interest

The authors declare that the research was conducted in the absence of any commercial or financial relationships that could be construed as a potential conflict of interest.

## Publisher’s Note

All claims expressed in this article are solely those of the authors and do not necessarily represent those of their affiliated organizations, or those of the publisher, the editors and the reviewers. Any product that may be evaluated in this article, or claim that may be made by its manufacturer, is not guaranteed or endorsed by the publisher.
